# Transient receptor potential channels as predictive marker and potential indicator of chemoresistance in colon cancer

**DOI:** 10.32604/or.2023.043053

**Published:** 2023-11-15

**Authors:** WEI HU, THOMAS WARTMANN, MARCO STRECKER, ARISTOTELIS PERRAKIS, ROLAND CRONER, ARPAD SZALLASI, WENJIE SHI, ULF D. KAHLERT

**Affiliations:** 1 The Fourth Clinical Medical College of Yangzhou University, Nantong Rich Hospital, Nantong, China; 2 Molecular and Experimental Surgery, Clinic for General-, Visceral-, Vascular and Transplant Surgery, Faculty of Medicine and University Hospital Magdeburg, Otto-von-Guericke University, Magdeburg, Germany; 3 Department of Pathology and Experimental Cancer Research, Semmelweis University, Budapest, Hungary

**Keywords:** Colon cancer, Transient receptor potential channels, Prognostic signature, Chemotherapy efficiency, TRPM5

## Abstract

Transient receptor potential (TRP) channels are strongly associated with colon cancer development and progression. This study leveraged a multivariate Cox regression model on publicly available datasets to construct a TRP channels-associated gene signature, with further validation of signature in real world samples from our hospital treated patient samples. Kaplan-Meier (K-M) survival analysis and receiver operating characteristic (ROC) curves were employed to evaluate this gene signature’s predictive accuracy and robustness in both training and testing cohorts, respectively. Additionally, the study utilized the CIBERSORT algorithm and single-sample gene set enrichment analysis to explore the signature’s immune infiltration landscape and underlying functional implications. The support vector machine algorithm was applied to evaluate the signature’s potential in predicting chemotherapy outcomes. The findings unveiled a novel three TRP channels-related gene signature (MCOLN1, TRPM5, and TRPV4) in colon adenocarcinoma (COAD). The ROC and K-M survival curves in the training dataset (AUC = 0.761; *p* = 1.58e-05) and testing dataset (AUC = 0.699; *p* = 0.004) showed the signature’s robust predictive capability for the overall survival of COAD patients. Analysis of the immune infiltration landscape associated with the signature revealed higher immune infiltration, especially an increased presence of M2 macrophages, in high-risk group patients compared to their low-risk counterparts. High-risk score patients also exhibited potential responsiveness to immune checkpoint inhibitor therapy, evident through increased CD86 and PD-1 expression profiles. Moreover, the TRPM5 gene within the signature was highly expressed in the chemoresistance group (*p* = 0.00095) and associated with poor prognosis (*p* = 0.036) in COAD patients, highlighting its role as a hub gene of chemoresistance. Ultimately, this signature emerged as an independent prognosis factor for COAD patients (*p* = 6.48e-06) and expression of model gene are validated by public data and real-world patients. Overall, this bioinformatics study provides valuable insights into the prognostic implications and potential chemotherapy resistance mechanisms associated with TRPs-related genes in colon cancer.

## Introduction

Colon adenocarcinoma (COAD) is the most common pathological subtype of colon cancer, accounting for more than 90% of cases [1]. Alarming global cancer statistics from 2020 indicate that colon cancer ranks as the second leading cause of cancer-related mortality, with approximately 0.94 out of 10.0 million cancer-related deaths attributed to this malignancy [2]. The therapeutic approach to colon cancer predominantly comprises a multifaceted strategy combining surgery, adjuvant chemotherapy, radiotherapy, and targeted therapy [3]. Despite significant advancements in clinical treatments for colon cancer, patient outcomes and overall prognosis remain poor, especially for those at advanced stages experiencing local recurrence or grappling metastasis, often due to chemotherapy resistance [4], tumor heterogeneity [5], and the like. The 5-year overall survival (OS) rate for colon cancer patients has been reported at a mere 63% [6], while for metastatic colon cancer, the figure dwindles to less than 15% [7]. Therefore, although extensive research has proposed a variety of promising new therapeutic targets, alongside the toolbox of established diagnostic and therapeutic indicative biomarkers the quest for novel prognostic markers and targets for overcoming chemotherapy resistance of colon cancer and to eradicate metastatic spread continues unabated in order to improve future patient care.

Transient receptor potential (TRP) channels are a unique family of ion channels discovered in 1969 [8]. Distinguished by remarkable cation selectivity and diversity of specific activation mechanisms from other ion channel families [9], this group now includes 27 known mammalian TRP proteins [10], categorized into six subfamilies based on distinct protein sequences: TRPC (Canonical), TRPV (Vanilloid), TRPM (Melastatin), TRPA (Ankyrin), TRPP (Polycystin) and TRPML (Mucolipin). These TRP proteins are widely distributed throughout the peripheral and central nervous systems, playing essential roles in the intracellular compartment and plasma membrane [11]. Extensive research has demonstrated the involvement of mammalian TRP channels in the production and regulation of nociception [9]. For instance, TRPV1 has emerged as a valuable target for pain management with great translational value [12,13]. Of note, investigating the tumor cell to nerve cell interaction has emerged as a promising area of research to develop new treatment strategies for a variety of cancers. Since members of the TRP family play pivotal roles in the communication of neuronal cells with its surroundings, we sought to screen for any indicative roles of TRP family members in the context of colon cancerogenesis. Furthermore, drugs targeting TRPV1, such as resiniferatoxin, have shown promise in alleviating severe adverse effects endured by cancer patients during chemotherapy and managing cancer-related pain [14]. As research progresses, TRP channels have garnered attention as potential targets for treating respiratory [15] and neurological diseases [16,17]. In addition, their association with tumor growth and cancer progression has led to the consideration of TRP channels as therapeutic targets for cancers, including metastatic cancers, where high TRP protein expression appears to play a role [18]. By regulating intra and extracellular Ca^2+^ concentration, TRP proteins can influence epithelial-mesenchymal transition induction via Ca^2+^-driven activation of PI3/AKT pathway and extracellular matrix (ECM) remodeling via regulating matrix stiffness through matrix metalloproteinases [19]. Consequently, targeting TRP channels has demonstrated significant clinical potential, with ongoing development of drugs to address a wide range of conditions, including eye disorders, sensory disorders, heart diseases, and neurological ailments [20]. Encouragingly, several compounds targeting TRPV1, TRPV3, TRPM8, and TRPA1 channels have progressed to clinical trials [21]. Supporting this, Pagano et al. [22] have shown that pharmacologically blocking TRPM8 in mice xenograft models inhibits the WNT/β-catenin signaling pathway, leading to reduced colon tumor growth. Therefore, strengthening TRP research, both in basic and clinical research, is indispensable for advancing novel cancer therapies, such as agonists and antagonists [23], along with identifying new biomarkers associated with TRP channels [24].

This study employed univariate and multivariate Cox regression analyses to construct a TRP proteins-related gene signature based on the publicly available COAD dataset from The Cancer Genome Atlas (TCGA). This signature was then comprehensively validated to assess its potential in predicting prognosis, immune features, and chemotherapy efficacy in colon cancer patients. Cancer cell on-target specificity was validated with real world samples from patients treated in our hospital. We present promising results that may pave the way for new ideas for innovative approaches to improve prognostic and treatment strategies, with a particular focus on addressing chemotherapy resistance in colon cancer.

## Materials and Methods

### Data obtaining and preparation

**Figure 1:**
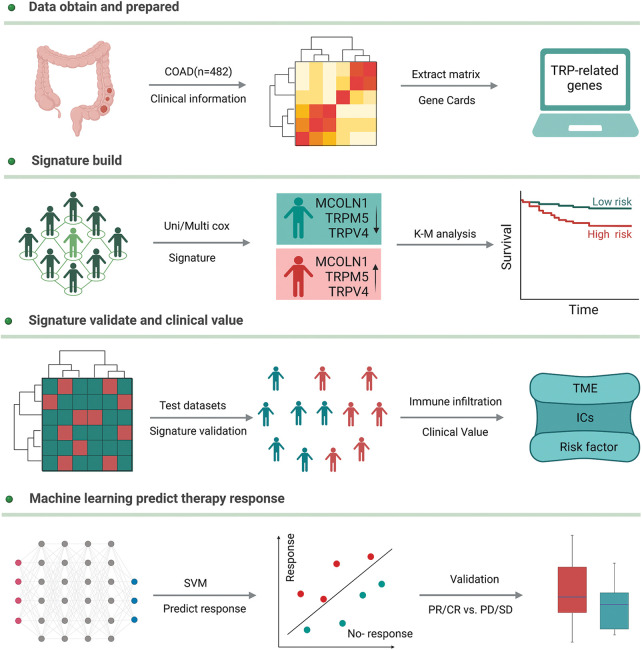
The detailed workflow of our study.

We downloaded the gene expression profile and clinical information of colon adenocarcinoma from the TCGA database (https://portal.gdc.cancer.gov/) and transferred the gene expression profile of Fragments Per Kilobase of exon model per Million mapped fragments (FPKM) to Transcripts Per Kilobase of exon model per Million mapped reads (TPM) style. 27 TRPs-related genes, obtained from the previous report, were extracted from the whole expression profile and used to construct a new matrix for continued analysis. The new expression profile was randomly split into two groups, according to the ratio of 7 to 3. 70% of samples were defined as a training group to build up the model and others were defined as a test group to validate the model robustness. In addition, the endpoint was defined as overall survival and samples with a survival time of fewer than 30 days were excluded. Patients who accepted chemotherapy with stable disease (SD), partial response (PR), or complete response (CR), were defined as sensitive, and others as resistant. These patients were similarly randomly split into two groups according to the ratio of 7 to 3, 70% of patients were used to test if the above signature could also predict chemotherapy efficacy, and 30% were used to validate. The detailed workflow is shown in Fig. 1.

For validation of tumor cell upregulation of candidate genes we applied mRNA sequencing data from tumor and non-tumor specimen, as stratified by German-board certified pathologist by histomorphological means, of our hospital treated patients (n = 6, ethical vote of the ethcal commission of the medical faculty of Magdeburg 33.01). All participants provided written informed consent.

### Cox regression to select model gene

We used univariate Cox regression to identify prognosis-related TRP genes and considered that the number of total genes was only 28. To avoid the loss of potentially important variables, we set significant criteria with a *p* value less than 0.1. Next step, we input all of the above-mentioned significant TRPs-related genes into the multivariate Cox regression model, setting new significant criteria with *p* value less than 0.05, to identify independent prognosis genes which were also called model genes.

### Signature construction and validation

In the training data set, the model gene expression and regression coefficients were used to build a signature to further calculate the risk score of each patient. The detailed formula is risk score = coef × A expression + coef × B expression + coef × C expression. After the signature build, according to the median value of risk score, patients were divided into a high-risk group and a low-risk group, Kaplan-Meier (K-M) analysis was then used to compare survival differences between these two groups, and area under the curve (AUC) value of receiver operating characteristic (ROC) were calculated to evaluate the model’s accuracy. For validation signature, the test data set went through the same formula calculation and conducted survival analysis and ROC test.

### Immune infiltration landscape of the gene signature

More and more, evidence shows that TRPs are associated with the tumor microenvironment (TME), so, we also estimated the difference in the level of immune cell infiltration between the two different groups of patients by the CIBERSORT algorithm. Furthermore, infiltration scores of 13 immune-related functional activities were evaluated in both groups by single-sample gene set enrichment analysis (ssGSEA). Finally, we revealed the relationship between the two risk groups and the efficacy of immune checkpoint inhibitor (ICI) therapy.

### Gene signature with chemotherapy resistance

To identify if the above signature were even suitable to predict chemotherapy efficacy, we use machine learning algorithms, and support vector machine (SVM). First, we included signature genes into the model and used 70% of patients as a training set, and 30% of patients as a test set to validate predictive effectiveness.

### Resistance TRP-gene selection, survival analysis, and mechanism exploring

We validated the signature gene expression difference between the resistant and sensitive group to detect a significantly different model gene that will be considered as the hub gene of drug resistance. Survival analysis of this gene was also performed in the resistance group. K-M survival analysis method will perform this procedure. To explore the candidate resistance mechanism, we analyzed the correlation between the hub gene and the multi-drug resistance gene, MDR1.

### Signature with clinical factor and validation in public datasets and hospital patients

Univariate and multivariate Cox regression models were used to analyze and judge whether Signature is an independent risk factor for COAD patients, while compared with other important prognosis-related clinical factors such as age, sex, body mass index (BMI), and TNM stage. In addition, we validate the model gene expression in public datasets and RNA seq data of our hospital patients.

## Results

### Three TRPs-related genes are independent prognosis factors

**Figure 2:**
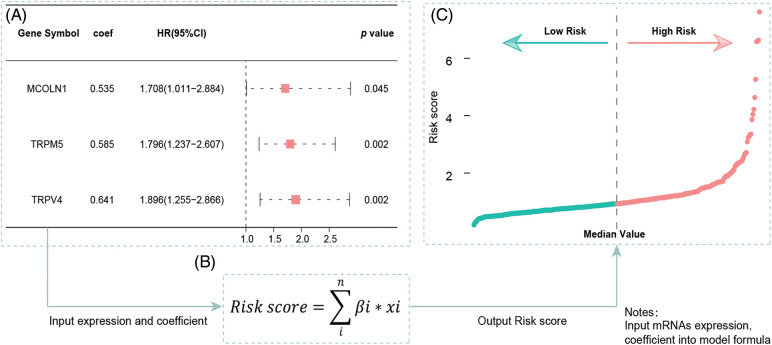
Multivariate Cox regression results and the signature building. (A) Multivariate Cox regression analysis built a three-gene (MCOLN1, TRPM5, TRPV4) prognostic signature. (B) The formula for calculating risk score. (C) Risk score distribution for patients in low- and high-risk groups from the TCGA-COAD cohort.

The univariate Cox regression model shows that a total of five TRPs-related genes could affect a patient's outcome; they are TRPV4, TRPM5, MCOLN1, TRPV2, and TRPV1 (Table 1). After multivariate Cox regression analysis, three TRPs-related genes (MCOLN1, TRPM5, and TRPV4) were identified as independent prognosis variables for COAD patients (Fig. 2A).

**Table 1:** Univariate Cox regression analysis of 27 TRPs-related genes with OS of colon cancer patients

Genes	HR	95% CI		*p* value
		Low	High	
TRPV4	2.294241459	1.550181327	3.395437542	3.30E-05
TRPM5	1.805044738	1.247105421	2.612599106	0.001745221
MCOLN1	2.075121031	1.286288111	3.347716001	0.002774194
TRPV2	1.357317463	0.984938137	1.870483663	0.061872403
TRPV1	12.94144668	0.742576626	225.5404174	0.07911233

Abbreviations: Transient receptor potentials; OS, overall survival; HR, hazard ratio (*p* < 0.01).

### Building of TRP gene signature and its validation for clinical prognostic value for colon cancer patients

We included the above-mentioned TRPs-related genes into the formula, according to coefficients and gene expression values to build a three TRPs-related gene signature (Fig. 2B). According to this signature, each patient will calculate a risk score, patients will be defined as high-risk group with a risk score more than the median value, while those will be defined as low-risk group (Fig. 2C).

**Figure 3:**
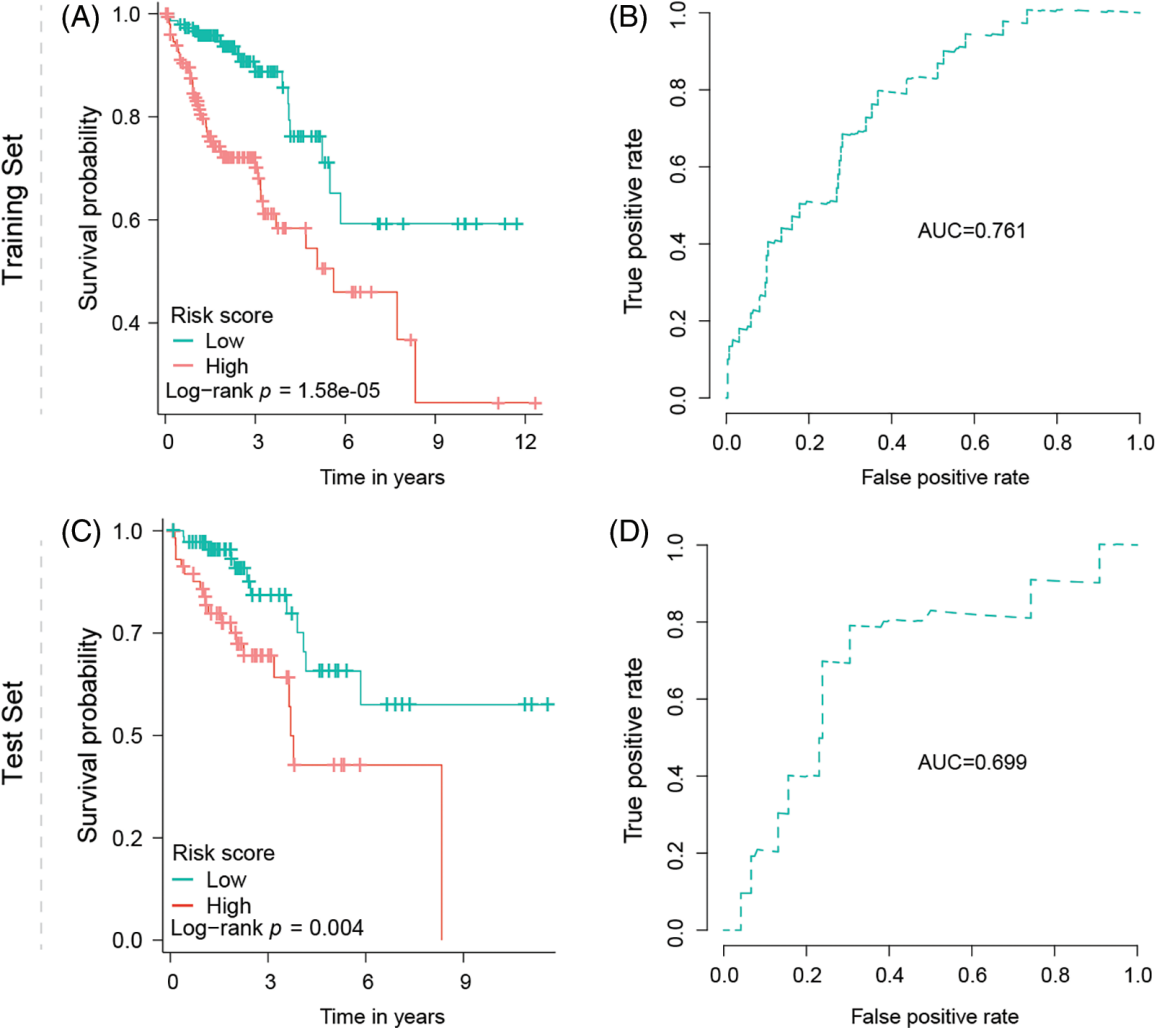
Signature validation in training and test dataset. (A) Kaplan–Meier survival analysis of OS between the low- and high-risk groups from the training dataset. (B) Predictability of the risk score signature predicting the OS in the training dataset indicated by AUC value. (C) Kaplan–Meier survival analysis of OS between the low- and high-risk groups from the test dataset. (D) Predictability of the risk score signature predicting the OS in the test dataset indicated by AUC value.

Survival analysis indicates low-risk group patients as significantly advantaged (*p* = 1.58e-05) over patients with high-risk gene signatures, regarding overall survival time (Fig. 3A). ROC analysis furthermore supports the signature’s predictive ability, with an area under the curve value (AUC) equal to 0.761 (Fig. 3B). For the test robustness of this signature, 30% of the patients were used as a test data set, once more validating that low-risk group patients have a better prognosis, compared with the high-risk group (*p* = 0.004), supported by an AUC of 0.699 (Figs. 3C, 3D).

### Tumors of TRP-High-risk score patients are associated with increased immune cell infiltration and altered immune checkpoint expression

**Figure 4:**
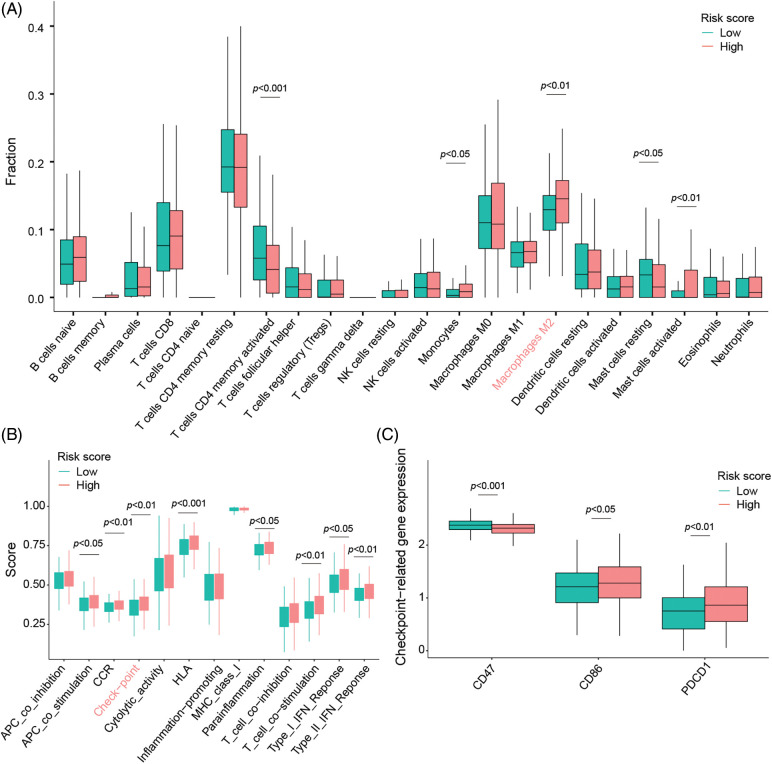
Signature evaluation in relationship to TME signals and activation of immune checkpoints. (A) Differences of 22 different types of tumor-infiltrating immune cells between low- and high-risk groups evaluated by the CIBERSORT algorithm. (B) ssGSEA scores evaluating differences in 13 immune-related functions between high-risk and low-risk groups. (C) The expression of immune checkpoints in low- and high-risk groups.

Immune infiltration analysis points out the high-risk group with more immune infiltration abundance in general especially highlighted by an increased M2 macrophage infiltration compared to patients with low-risk status (Fig. 4A). In addition, immune-related functional analysis displays that this signature is associated with immune checkpoints, as well as higher immune scores (Fig. 4B) making the signature even more clinically relevant as the high-risk group patients could potentially benefit from an immune checkpoint inhibitor therapy (Fig. 4C) caused by an increased CD86 and PD-1 (also called PDCD1) expression profile.

### Three TRPs-related gene signature predicts adjuvant chemotherapy efficacy

**Figure 5:**
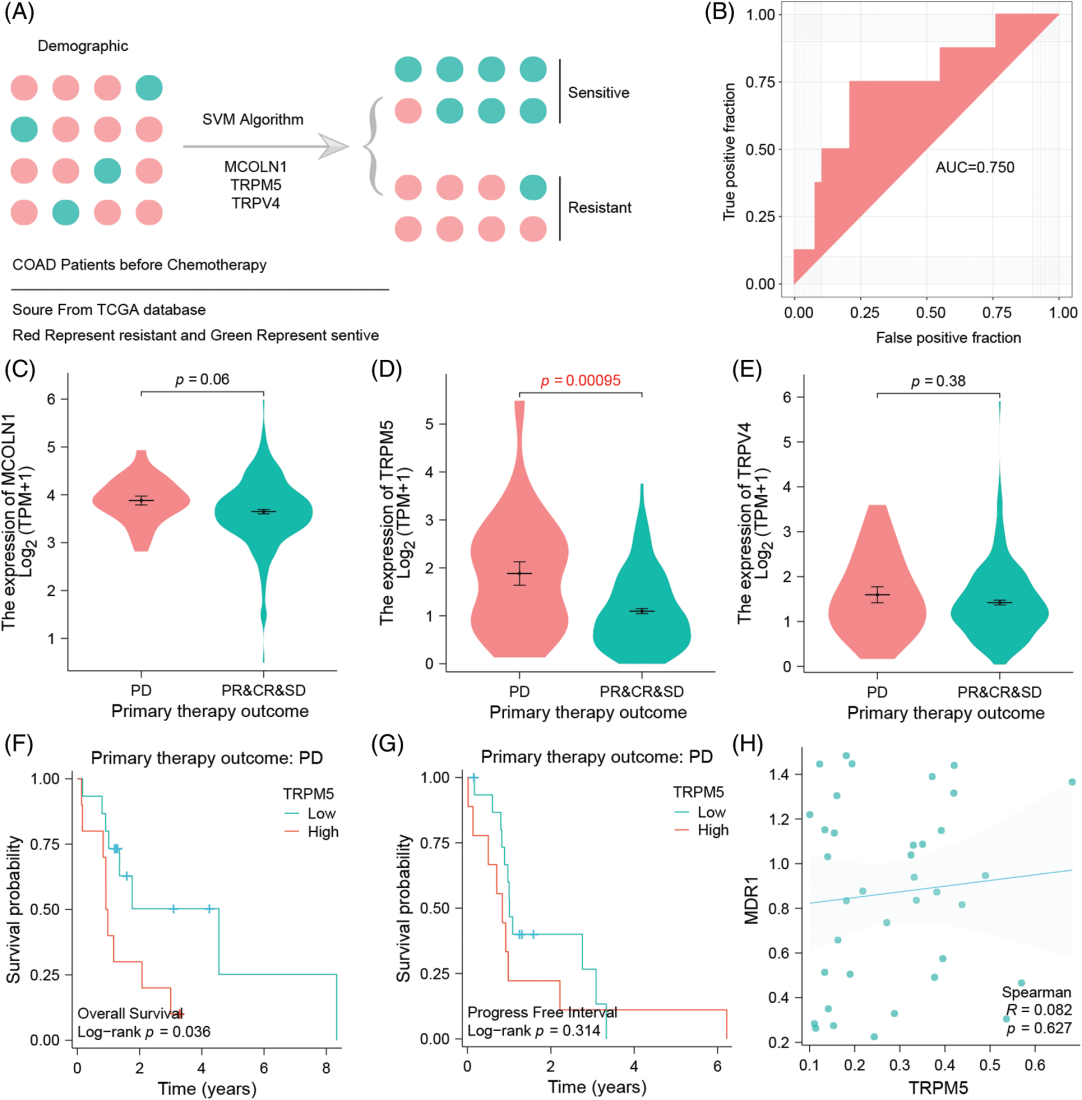
Three TRPs-related genes signatures could predict the efficacy of clinical standard-of-care chemotherapy. Chemo-resistant group is defined by progressive disease (PD), whereas chemo-sensitive tumors combine responding cases of partial response (PR), complete response (CR), or stable disease (SD) (A) SVM model to assess the signature’s efficacy for predicting chemotherapy. (B) ROC curve to verify the accuracy of the SVM model. (C) The difference of MCOLN1 gene expression between the chemoresistant group and chemosensitive group. (D) The difference of TRPM5 gene expression between the chemoresistance group and chemosensitivity group. (E) The difference of TRPV4 gene expression in dependency of the chemo resistance level of the tumor. (F) Association between TRPM5 expression and overall survival in the drug-resistant group of COAD patients. (G) Association between TRPM5 expression and progression-free interval in the drug-resistant group of COAD patients. (H) Correlation of TRPM5 gene expression and MDR1 gene expression.

Adjuvant chemotherapy treatment is a baseline treatment option in COAD patients from UICC II-IV. A major problem of this treatment is the development of chemotherapy resistance, making the detection of sensitive patients by analyzing their gene signature a promising method to improve the patient’s outcome (Fig. 5A). Here, we included three TRP-related genes in the machine learning model, SVM, to validate if these genes could moreover be used to predict chemotherapy efficacy. Machine learning is showing that in the test data set, the model’s AUC is equal to 0.75. These results suggest that the three genes have a good predictive ability to identify and differentiate between sensitive and resistant populations (Fig. 5B).

### TRPM5 is a hub gene of resistance and is associated with the patient’s survival performance

Expression analysis of three TRPs-related gene signatures shows that, compared with resistance and sensitivity, the three genes are expressed highly in the resistance group, although MCOLN1 and TRPV4 genes show no significance between those groups (*p* = 0.06 *vs*. *p* = 0.38). In contrast, TRPM5 is highly and significantly expressed in the resistance group, which is why we defined it as the hub gene of resistance (*p* = 0.00095) (Figs. 5C–5E). Survival analysis additionally shows that in the resistant group, high expression TRPM5 means poor overall survival which significantly differs from the low expression group (*p* = 0.036) (Fig. 5F). However, the trend can also be observed in the progress-free interval, but there is no significance between the high and low groups (*p* = 0.314) (Fig. 5G).

### TRPM5 expression is positively correlated with the MDR1 gene

We have demonstrated that TRPM5 is a hub gene of resistance, considering that the ion channel could potentially play an important role in the formation of chemoresistance. This is why we tried to discover that specific mechanism of TRPM5 by analyzing TRPM5 expression with the classic MDR1 gene. Expression is indeed correlated with MDR1 (R = 0.082), but we were not able to find any significance (*p* = 0.627) (Fig. 5H). Because MDR1 is able to decrease drug concentration by efflux mechanism, we may infer that TRPM5 is an Ion channel marker; although efflux anti-neoplastic drugs could lead to drug resistance, validation of this hypothesis has to be carried out by further basic experiments.

### Three TRPs-related genes signature is an independent prognostic factor

It is common sense that clinical variables could affect a patient’s outcome, such as age, sex, BMI, tumor stage, and many more. For that reason, we combined these factors with our risk score and included a univariate and multivariate Cox regression model. The result: compared with other clinical variables, our risk score is an independent prognostic factor for COAD patients, which means that this risk score is able to give a prognostic and valid benefit in diagnostics without being affected by the patient’s underlying clinical characteristics. (*p* = 6.48E-06) (Suppl. Table S1).

### Three TRPs-related gene expression difference between normal and cancerous colon tissues

**Figure 6:**
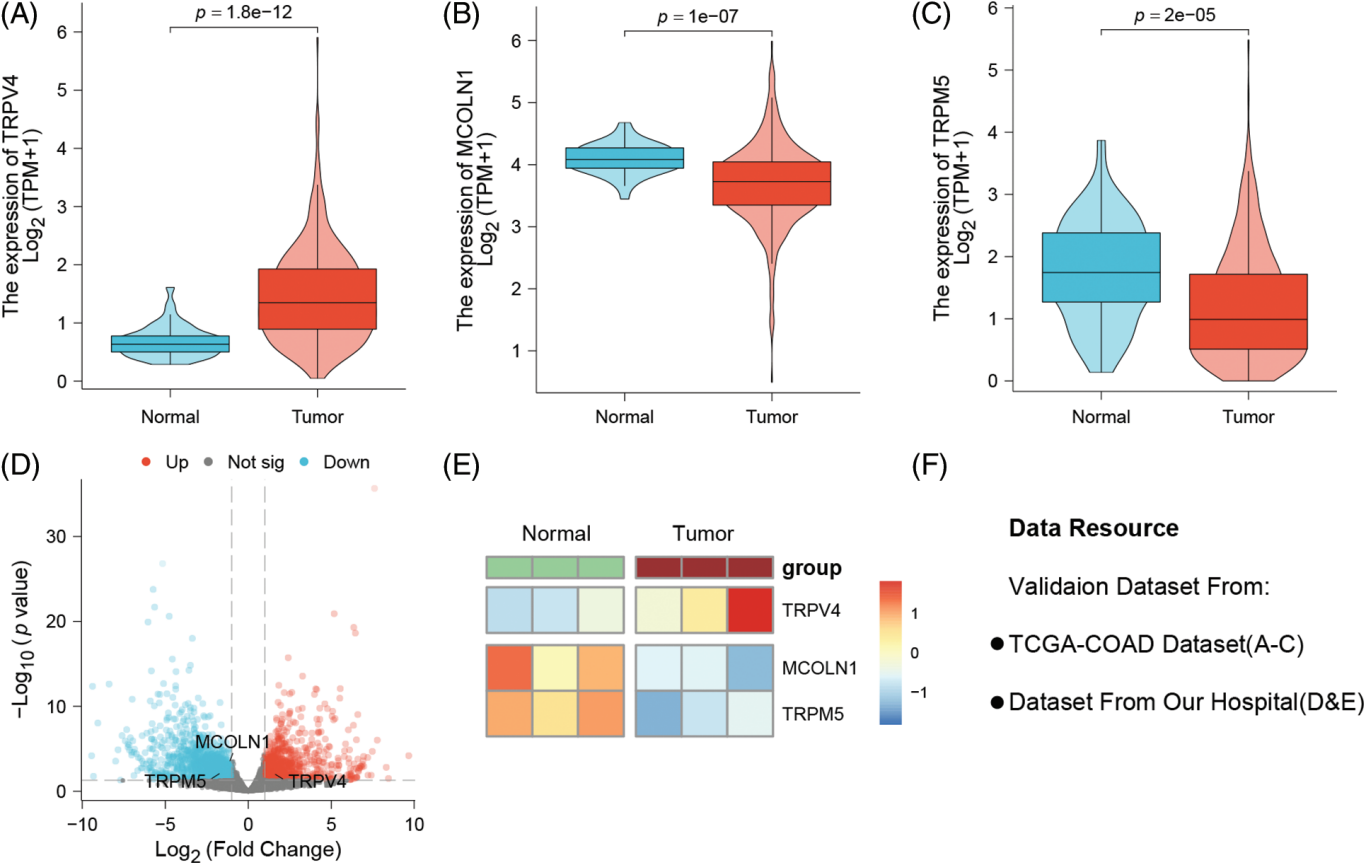
Probing the three model genes for their expression differences between normal tissues and colon cancer tissues by public data and real-world patients. (A) TRPV4 is strongly overexpressed in colon cancer tissues (*p* = 1.8e-12). (B) MCOLN1 and (C) TRPM5 downregulated in cancer tissues (*p* = 1e-07; *p* = 2e-05). (D) Our own hospital patient samples: The volcano plot of different expression genes between normal and tumor tissues. (E) The heatmap of three model genes between normal and tumor tissues. (F) Note to explain clearly of data resource.

According to the public data analysis results show that TRPV4 is highly expressed in colon cancer tissues while it is low expressed in normal tissues (*p* = 1.8e-12) (Fig. 6A), However, MCOLN1 and TRPM5 are both highly expressed in normal tissues, while low expressed in tumor group (*p* = 1e-07, *p* = 2e-05; respectively) (Figs. 6B and 6C). In real-world study, we collected three colon cancer patients’ tissues and related paracancerous tissue conducting different expression analysis. The results show that TRPV4, MCOLN1,and TRPM5 are expressed differently between normal and tumor tissues (Figs. 6C and 6D). The Fig. 6F as note to explain clearly data resource of validation datasets.

## Discussion

Colon cancer, a prevalent form of malignant gastrointestinal cancer with a grim global prognosis [25], necessitates dedicated clinical research to identify reliable prognostic biomarkers and patient outcomes [26]. Moreover, extensive studies over the past decades have underscored the pivotal role of TRP channels in various aspects of tumor growth and cancer progression [27]. However, our understanding of TRP channels’ precise role and molecular mechanisms in colon cancer remains limited. In this study, we have developed a gene signature based on three TRP channel genes. To evaluate its predictive efficiency, we thoroughly investigated and validated this signature using both training and test datasets from the TCGA-COAD cohort. Additionally, we explored the immune cell infiltration characteristics and the expression of immune checkpoint molecules associated with the signature. To better assess the clinical prognostic value of this signature, we performed an independent prognostic analysis by integrating the signature’s risk score with clinical variables. Furthermore, we employed machine learning algorithms to assess the signature’s capability to predict chemotherapy outcomes in colon cancer patients and identified a hub TRP channels-related gene linked to chemoresistance.

We initially screened five genes associated with OS of COAD patients using a univariate Cox regression model. Then, we constructed a gene signature based on three TRP channel genes, MCOLN1, TRPM5, and TRPV4, using a multivariate Cox regression model. These three genes were selected as optimal model genes through stepwise variable selection using the Akaike information criterion (AIC) by maximizing the concordance index and minimizing AIC values [28,29]. Upon successful signature development, we plotted the K-M survival curve and ROC curve based on the training dataset to evaluate the signature’s clinical utility and predictive efficacy, respectively. To ensure the robustness of the signature [30], we validated the signature’s predictive efficacy using an internal test dataset. The survival analysis results demonstrated the signature's strong prognostic value, effectively distinguishing high-risk from low-risk groups, with improved overall survival in the latter. Normally, AUC values of the ROC curve range from 0.5 to 1, with values greater than 0.7 indicating good predictive accuracy and reliability in practice [31]. In our study, the AUC value of the ROC curve in the training dataset was 0.761, signifying excellent predictive performance of the TRP channels-related gene signature. Although the AUC value decreased slightly (AUC = 0.699) in the internal test dataset, it remained close to 0.7, indicating the signature’s robustness and reliability. Furthermore, independent prognostic analyses revealed that this three-gene signature was an independent prognostic factor for predicting OS of colon cancer patients, unaffected by patients’ clinical features. In general, our current study suggests that the TRP channel-related gene signature holds promise for stratifying colon cancer patients based on prognostic risk.

The signature we constructed consists of three genes (MCOLN1, TRPM5, and TRPV4), all identified as independent prognostic and risk factors for colon cancer through the multivariate Cox regression model. MCOLN1, also known as TRPML1, belongs to the TRP channel family and is specifically localized in lysosomes [32,33]. It plays a crucial role as a Ca^2+^ conductance channel in the lysosomal membranes, facilitating lysosomal Ca^2+^ release and regulating Ca^2+^ influx from lysosomes to the cytosol [34]. TRPML1 is a reactive oxygen species (ROS) sensor in lysosomes, coordinating an autophagy-dependent negative feedback program to mitigate cellular oxidative stress [35]. MCOLN1/TRPML1 also regulates autophagy through multiple pathways, including activating calcineurin to dephosphorylate TFEB, thereby promoting autophagy [36]. Moreover, MCOLN1/TRPML1 finely controls oncogenic autophagy in cancer by mediating zinc influx into the cytosol [37]. Downregulated MCOLN1 has been associated with decreased lysosome-autophagy activity and suppressed tumor progression in non-small cell lung cancer (NSCLC) [38]. In malignant melanoma and glioma cell lines, MCOLN1/TRPML1-induced autophagy inhibition has been shown to impede cancer metastasis via the ROS-mediated TP53/p53 pathway [39]. Thus, MCOLN1 has great potential in inhibiting cancer progression and represents a promising drug target for cancer treatment. TRPM5 is a monovalent-specific cation channel activated by Ca^2+^, functioning in taste receptor cells [40] and responding to warm temperatures [41]. TRPM5 is expressed in various tissues, including olfactory neuron subpopulations, the respiratory system, pancreatic islets [42,43], and the gastrointestinal tract, such as the stomach, small intestine, and colon [44,45]. In human colonic cancer cells (HT29-18N2), TRPM5 channels have the potential as pharmacological targets for managing mucus-associated pathologies, such as cystic fibrosis [46], given their roles in regulating Ca^2+^-mediated mucin 2 and MUC5AC secretion [47]. TRPM5 agonists have shown promise in promoting rodent gastrointestinal prokinetic activity [48]. High TRPM5 mRNA expression has been associated with poor OS in both gastric cancer and melanoma patients [49].

TRP vanilloid 4 (TRPV4) is a mechanosensitive ion channel activated by mechanical and biochemical stimuli. It is commonly expressed in a variety of cell types, including macrophages and myofibroblasts [50], and plays a significant role in many physiological and pathophysiological processes such as joint diseases [51], pulmonary inflammatory diseases [52], and multiple cancers [53,54]. In colon cancer, Liu et al. [55] have shown that inhibiting TRPV4 suppressed colon cancer development by activating the PTEN pathway, thereby impairing the AKT-mTOR signaling cascade. Zhang et al. [56] also uncovered that inhibiting TRPV4 hindered colorectal cancer migration and invasion by suppressing the epithelial-mesenchymal transition process. Furthermore, TRPV4 antagonists have shown therapeutic potential in experiment animal models for a range of conditions, including heart failure, edema, pain, gastrointestinal disorders, lung diseases, and various cancers [57]. In addition, at least seven new TRPV4 agonists or antagonists have been reported to date [58]. TRPV4 has also shown promise in diagnostics, playing an important role in predicting early lymph node metastasis and poor OS in gastric [59] and ovarian adenocarcinoma [60]. Overall, TRPV4 has emerged as a potential target for treating several human diseases [61]. Taken together, the previous studies mentioned above have shown that these three genes in the signature are widely involved in multiple diseases, including cancer progression, such as colon cancer. Thus, they are expected to be novel biological markers and therapeutic targets for colon cancer. Meanwhile, given the limited prior research on these three genes’ prognostic and oncogenic molecular mechanisms in colon cancer, our study provides new evidence that enriches our understanding of their prognostic functions and molecular mechanisms in colon cancer.

A substantial body of studies have now firmly established the crucial role of tumor microenvironment in tumor progression. Tumor-infiltrating immune cells, such as CD8^+^ T cells, CD4^+^ T cells, and macrophages, are important components of the tumor microenvironment and can affect tumor progression and patient prognosis [62]. M2 macrophages, a predominant subset of macrophages, exhibit immunosuppressive activities and pro-tumoral effects in the tumor microenvironment [63]. That implies that M2 macrophages contribute to creating an immunosuppressive tumor microenvironment, fostering cancer development and metastasis [64]. Macrophages infiltrating the tumor microenvironment are also known as tumor-associated macrophages, with M2 macrophages being the predominant phenotype [65]. Zhang et al. [66] have demonstrated that tumor-associated macrophages promote colon cancer cell migration and metastasis. Additionally, Hu et al. [67] reported a correlation between high levels of M2 macrophage infiltration and poor prognosis in colon cancer patients. Our findings are consistent with previous studies, as we observed increased M2 macrophage infiltration in the high-risk group, implying that patients in this group represent an immunosuppressive subtype associated with a poor prognosis.

Tumor immunotherapy has emerged as a groundbreaking development in biomedicine, intending to eliminate tumors by reshaping the tumor immune microenvironment and activating the body's normal anti-tumor immune response [68]. Immune checkpoint inhibitors (ICIs), including programmed cell death-1 (PD-1), programmed death-ligand 1 (PD-L1), and cytotoxic T-lymphocyte antigen 4 (CTLA4) inhibitors [69], are a modality of tumor immunotherapy. Besides, ongoing research is exploring new antibodies or inhibitors targeting other immune checkpoints, such as CD47 [70], LAG3, and TIM3 [71]. Over the past few years, ICIs have demonstrated great promise in treating various solid tumors, including melanoma, non-small cell lung cancer, and prostate cancer [72]. Consequently, research using ICIs to target colon cancer is advancing rapidly to improve the clinical outcomes of colon cancer patients. In colorectal cancer, the clinical use of PD-1/PD-L1 inhibitors is currently based on mutation patterns. Notably, the United States Food and Drug Administration (FDA) has approved using PD-1/PD-L1 inhibitors pembrolizumab and nivolumab for patients with dMMR-MSI-H colorectal cancer [73]. In our study, we observed differences in the expression levels of various immune checkpoint molecules, including PDCD1, CD86, and CD47, between the high and low-risk groups, indicating that colon cancer patients in the two risk groups may exhibit different responses to ICIs. Specifically, our results revealed higher PDCD1 and CD86 levels but lower CD47 levels in the high-risk group, suggesting that colon cancer patients in the high-risk group may respond better to anti-PD-1 and anti-CD86 inhibitors, while those in the low-risk group may benefit more from anti-CD47 inhibitors.

Chemotherapy resistance remains a significant obstacle to effective cancer treatment [74] and presents one of the main clinical challenges in colon cancer management [4]. SVM is a supervised machine learning algorithm for tasks such as classification and regression and has been successfully applied in cancer genomics [75]. Hence, we employed SVM to evaluate the predictive efficacy of the three TRP channel-related-gene signature concerning chemotherapy outcomes of colon cancer patients. We calculated the AUC of the ROC curve in the test dataset for internal validation of our prediction results. Our data revealed an AUC of 0.75 in the test dataset, suggesting that the three genes (MCOLN1, TRPM5, and TRPV4) possess robust predictive ability for distinguishing drug-sensitive and drug-resistant populations. Subsequently, in our quest to unveil potential drug-resistant targets for colon cancer, we identified a hub gene based on the expression differences of these three genes in the TRP channel genes-related signature between the drug-resistant and drug-sensitive groups and performed the K-M survival analysis to validate this hub gene. Our findings substantiated TRPM5 as a pivotal drug-resistant hub gene associated with OS of COAD patients. This association was evident from TRPM5's heightened expression in the drug-resistant group and its association with a poor OS rate of COAD patients. Dysfunction of the TRPM5 channel has been linked to a range of disease states, including diabetes, intestinal infections, and inflammatory responses [76]. Additionally, TRPM5 is recognized as a potential pharmacological target for the development of novel insulin secretagogue [77]. TRPM5 channel serves as a chemosensory TRP channel involved in the signaling of chemical substances [44]. Although research into the role of TRPM5 in cancer chemoresistance and the development of TRPM5-targeting drugs is limited, a small molecule compound targeting TRPM5, identified by Virginio et al. [78], has demonstrated substantial promise as a novel drug targeting several pathological conditions. This marks the initial stage of developing effective selective TRPM5 openers or positive modulators. To further explore the mechanism underlying the chemoresistance associated with TRPM5 gene for colon cancer, we unveiled a positive correlation between the expression of TRPM5 and the multi-drug resistance gene, MDR1. This observation underscores the potential of TRPM5 as a viable drug target for combating drug resistance in colon cancer. Since there is no statistical significance in expression values of MCOLN1 or TRPV4 in chemo resistance group as compared to chemo sensitive group, but the assembly of the presented three gene panel risk score is urging their relevance in contributing to disease malignancy, we speculate that those genes prominently insect in other colon cancer cell processes than those regulating drug resistance. Nonetheless, additional studies are needed to validate our hypothesis and unravel the role of TRP channels, particularly in developing chemotherapy resistance in COAD. One practical approach that could significantly impact patient outcomes involves integrating immunohistochemical staining for TRPM5 into standard pathological diagnostics before sequencing patient material. This cost-effective and straightforward procedure could effectively pre-screen patients and guide treatment decisions, thereby enhancing the overall outcome by categorizing patients into subgroups that might benefit from our TRP channel-related gene signature.

In summary, our study is the first to introduce a novel three-gene signature based on TRP channel-related genes, shedding light on the potential of TRPM5 as a target for combatting chemoresistance in colon cancer. However, certain limitations in our study need to be improved. Firstly, validating the clinical utility of this signature within TCGA's internal test dataset is insufficient. Thus, further external validation involving prospective studies and real-world colon cancer cohorts is essential to establish the prognostic value of this signature model. Secondly, although we have uncovered an association between TRPM5 levels and chemotherapy resistance in colon cancer, the specific mechanisms remain to be explored experimentally.

## Conclusions

We have successfully constructed and validated a novel gene signature comprising three TRP channel genes (MCOLN1, TRPM5, and TRPV4) based on the TCGA-COAD dataset. This signature demonstrates strong capabilities to predict colon cancer patients’ prognosis, immune features, and chemotherapy efficacy. Furthermore, we have identified TRPM5 as a pivotal hub gene associated with chemoresistance, offering potential as a molecular target for overcoming chemoresistance in colon cancer patients. The findings advance our understanding of the prognostic and chemoresistance mechanisms related to TRP channel-related genes in colon cancer. Wet lab experiments addressing the functional roles of selected TRP genes in colon cancer, such as possible interaction with the neural microenvironment, are warranted to offer any mechanistic insight related to our hypothesis.

## Supplementary Materials



## Data Availability

The datasets presented in this study can be obtained from the corresponding author.

## Abbreviations

COAD: Colon adenocarcinoma
TRP: Transient receptor potential
K-M: Kaplan-Meier
AUC: Area under the curve
ROC: Receiver operating characteristic
ICI: Immune checkpoint inhibitor

## Supplementary Materials

The supplementary material is available online at https://doi.org/10.32604/or.2023.43053.
